# Sustainable fashion: eco-friendly dyeing of wool fiber with novel mixtures of biodegradable natural dyes

**DOI:** 10.1038/s41598-022-25495-6

**Published:** 2022-12-05

**Authors:** Lina Lin, Tiancheng Jiang, Lexin Xiao, Md. Nahid Pervez, Xiaobo Cai, Vincenzo Naddeo, Yingjie Cai

**Affiliations:** 1 Hubei Provincial Engineering Laboratory for Clean Production and High Value Utilization of Bio-Based Textile Materials, Wuhan Textile University, Wuhan, China; 2 Engineering Research Centre for Clean Production of Textile Dyeing and Printing, Ministry of Education, Wuhan Textile University, Wuhan, China; 3 Sanitary Environmental Engineering Division (SEED), Department of Civil Engineering, University of Salerno, 84084 Fisciano, Italy; 4 TST Group Holding Ltd., Guangzhou, 510620 China

**Keywords:** Materials science, Biomaterials

## Abstract

Natural materials, especially natural colorants, have achieved global prominence and might be regarded as an environmentally beneficial alternative to hazardous synthetic dyes. The color limitation of natural dyes hinders their application in textiles. The present work aims to prepare more color shades of wool yarns via dyeing with ternary natural dye mixtures without adding mordants. In this study, a sustainable dyeing approach for wool yarn was evaluated with three natural dyes, madder red (MR), gardenia blue (GB), and gardenia yellow (GY), by following an industrial dyeing procedure in the absence of a mordant. In the beginning, a preliminary assessment of dye stabilities was carried out, and it was found that the three natural dyes were sensitive to temperature and acid (degradation tendency). Then, the dyeing behavior was systematically evaluated, including a single natural dye, a binary natural dye mixture, and a ternary natural dye mixture. The results of wool yarn dyeing with a single natural dye show that the dye exhaustion percentage (E%) of MR, GY, and GB was in the ranges of 78.7–94.1%, 13.4–44.1%, and 54.8–68.5%, respectively. The dyeing results of wool yarns dyed with binary and ternary natural dye mixtures (a color triangle framework of dyed wool yarn) were characterized by colorimetric values (L*, a*, b*, C*, h, and K/S), and are presented to enlighten various colorful shades. Finally, color uniformity and colorfastness tests confirmed the vital contribution of natural dyes toward wool yarn coloration. Particularly, colorfastness to washing confirmed the stability of natural dyes with reference to the lower amount of dyes released into the effluent, which is beneficial for the environment. Overall, this study provides a good background for enhancing the industrialization trend of natural dyes by modulating their dyeing scheme.

## Introduction

Synthetic dyes are commonly used for the coloration of textiles owing to their huge hues and easy application. However, the synthetic dyes and auxiliaries present in dyeing wastewater discharged into the environment cause toxic and allergic reactions^1–7^ to creatures, resulting in adverse effects on the ecosystem. With an increase in the concern on environmental issues, the use of natural dyes for textile coloration has recently increased owing to their biodegradability and compatibility with the environment^8–10^. The natural dyes not only color but also provide beneficial properties to the textiles^11,12^, such as antibacterial activity, antioxidant activity, and UV resistance. However, the disadvantages of low dye exhaustion percentage and fixation efficiency, especially weak light fastness, hinder its wide application^13–15^.

To overcome the inherent drawbacks of natural dyeing on a larger scale, mordants (metallic salts)^16,17^ such as aluminum potassium sulfate (KAl(SO_4_)_2_), stannous chloride (SnCl_2_), potassium dichromate (K_2_Cr_2_O_7_), and sodium chromate (Na_2_CrO_4_) are extensively added in dyeing by pre, simultaneous, or post-mordanting pattern^18,19^ to form complexes between the natural dye and fiber. After complexation, the natural dye is stably precipitated in fiber^20^, promoting dye exhaustion, fixation, and colorfastness to washing and rubbing^21–23^. However, the residual heavy metal ions of mordants in the discharge are harmful to the environment^24,25^. Besides, the color hue of natural dye is shifted after complexing with mordants, and different mordants result in different color changes, causing problems in controlling the color stability in batch production^26,27^.

Natural dyes are encouraged to be used in textile dyeing^20^ owing to their benefits to the ecosystem. However, the source of natural colorants limits their industrial application because the harvest time is dependent on the season^28,29^. Although the color range of natural dyes covers red, olive, burgundy, green, yellow, brown, blue, and black from the plant origin of bark, roots, leaves, fruits, and flowers^30,31^, it is still not comparative with that of synthetic dyes. In other words, the color hue of natural dye also challenges a natural dyeing product. In dyeing textiles with a synthetic dye, the color shade of a substance is generally prepared with ternary dye mixtures; hence, huge color shades of textiles are achieved. To overcome the color limitation of natural dyes, dyeing textile with ternary natural dye mixtures is a practical way to prepare more color shades of textiles. However, notably the addition of mordants in dyeing cause a color shift, and different natural dyes may need different mordants^32^. Thus, it is necessary to eliminate the addition of mordants in natural dyeing.

This study presents a new insight into the sustainable dyeing of wool yarn with mixtures of madder red (MR), gardenia blue (GB), and gardenia yellow (GY) dyes to prepare different color shades (color triangle) with various dye mass ratios without the addition of mordants. Dyeing with a binary and ternary mixture of natural dyes is still limited in natural dyeing plants^33^. In this study, not only colorful wool fibers were successfully produced using natural dye mixtures with satisfactory wash colorfastness, but also the research guidance on the coloration of fiber with natural dye mixtures is provided.

## Experimental

### Materials

**Figure 1:**
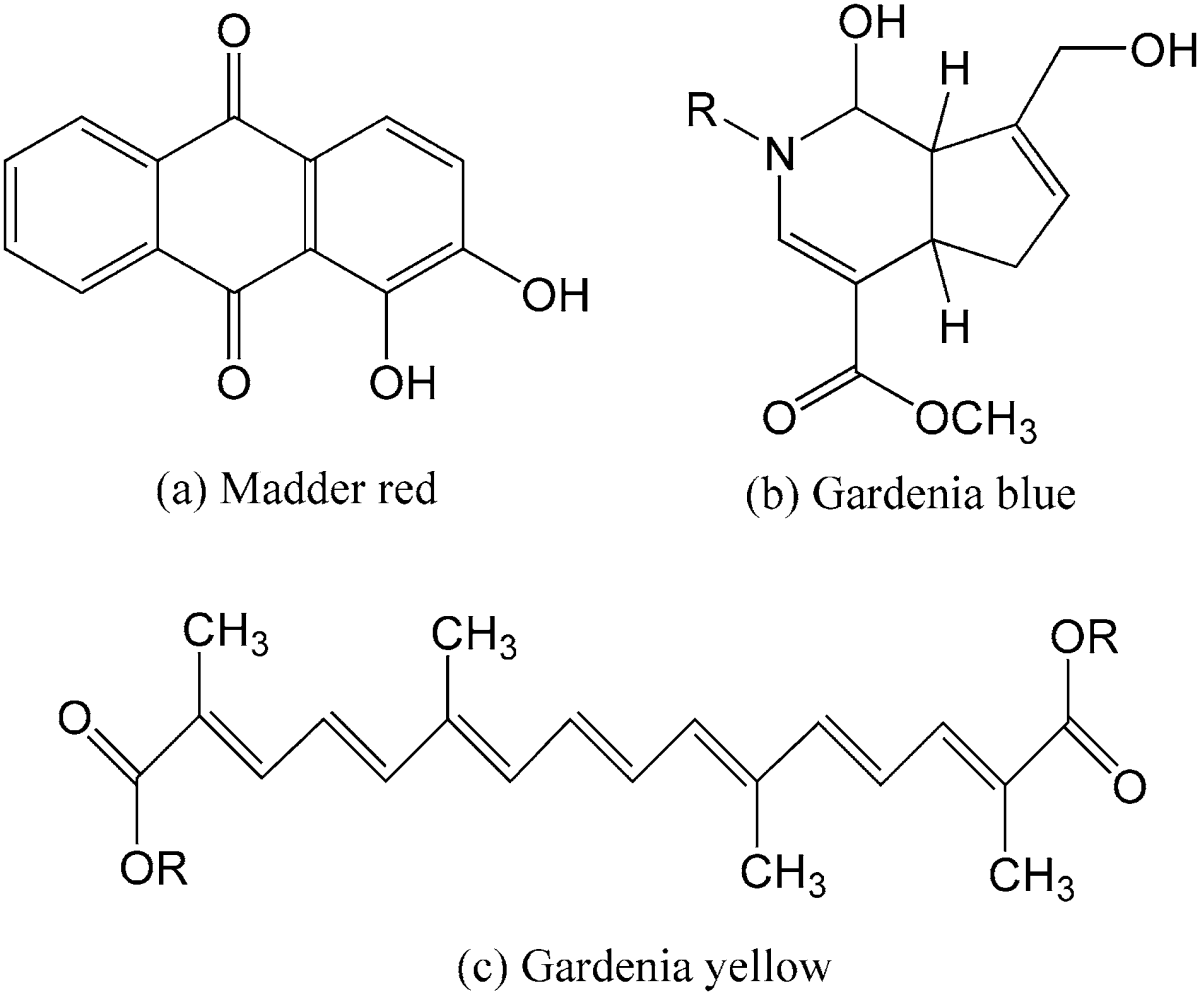
Chemical structures of main components of (**a**) madder red, (**b**) gardenia blue, and (**c**) gardenia yellow dyes.

Scored wool yarn (48 Nm/2, 100%) was a gift from a local dyeing plant. The MR dye was obtained from Zhongda Hengyuan Biotechnology Stock Co., Ltd. (China). The GB and GY dyes were obtained from Wuhan Green Food Biological Engineering Co. Ltd. (China). The chemical structures of the main components of MR^34^, GB^35,36^, and GY^37^ are shown in Fig. 1. Sodium acetate trihydrate (AR) and acetic acid (AR) were purchased from Sinopharm Chemical Reagent Co., Ltd (China). Nonionic detergent (Luton 500) was purchased from Dalton UK Company (Shanghai, China).

### Stability of natural dyes

To study the thermostability of these three natural dyes, various acidic buffer solutions with pH 3–6 were prepared using sodium acetate trihydrate and acetic acid in deionized water. The dye was added to the acidic buffer solution to prepare a dye solution with a concentration of 80 mg L^−1^ at pH 3–6. Subsequently, the dye solution was heated at a rate of 1 °C min^−1^ from 30 °C to a target temperature (60–100 °C), and then maintained at this level for a period (110–150 min) to make 180 min of total treatment time. The treatment process was completed in a rotary infrared radiation laboratory-dyeing machine (Automatic Prototype, Model: A-12, AQUA, China). The dye solution at various treatment periods was measured using a UV–Vis spectrophotometer (Cary 100, Agilent Technologies, Australia)^38^. The degradation percentage of dye (D%) was calculated using Eq. (1), where *A*_0_ and *A*_1_ are the light absorbance of the dye solution at the maximum absorbance wavelength (λ_max_) before and after treatment.

1 $$D\mathrm{\%}=\frac{{A}_{0}-{A}_{1}}{{A}_{0}}\times 100\%$$

### Dyeing of wool yarn

**Figure 2:**
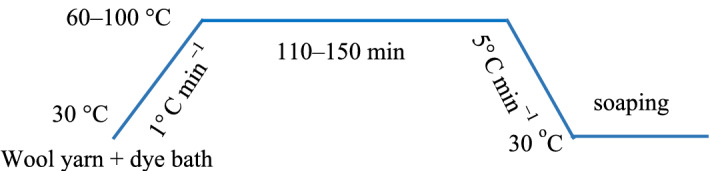
Dyeing of wool yarn with natural dye.

**Figure 3:**
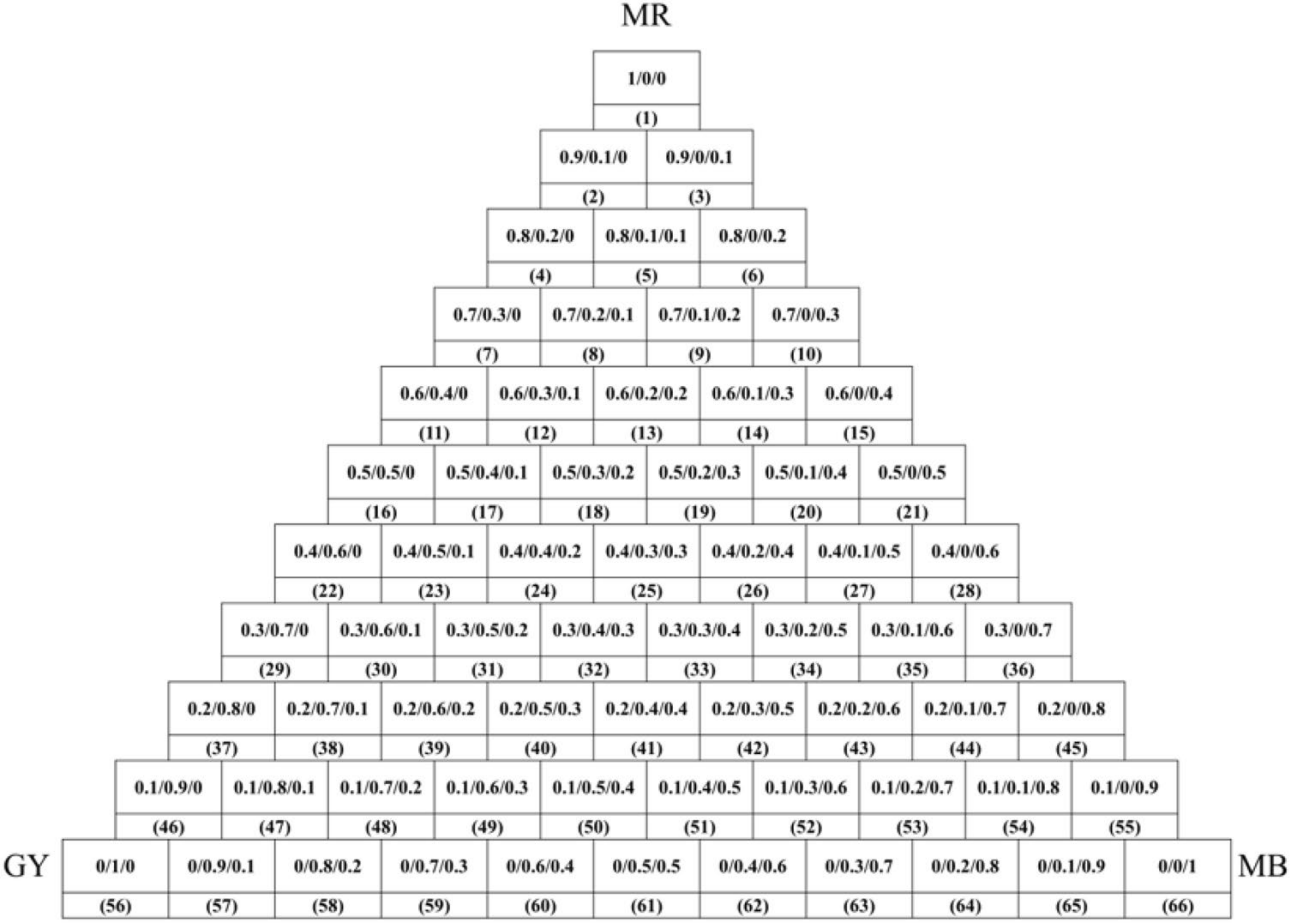
Color triangle of wool yarn dyeing using MR, GY, and GB dye mixtures with various dye mass ratios.

In dyeing with a single natural dye, the wool yarn (2 g) was dyed with various 2% o.m.f (on the mass of fiber) of natural dye in an acidic buffer solution at pH 3–6 with a liquor ratio of 25:1 at 60–100 °C for 180 min in a rotary infrared radiation laboratory-dyeing machine (Automatic Prototype, Model: A-12, AQUA, China)^39^. The dyeing processes are shown in Fig. 2. In dyeing with natural dye mixtures, the wool yarn (2 g) was dyed with 3% o.m.f of dye mixtures (varying weight ratios are shown in Fig. 3) at pH 3 and 90 °C with a liquor ratio of 25:1 and maintained at 90 °C for 40 min. After dyeing, the dyed sample was squeezed by hand to remove the excess dye solution in the wool yarns, followed by drying in an oven. Subsequently, the dried sample was treated in a soap solution containing 2 g L^−1^ of nonionic detergent at 95 °C for 15 min at a liquor ratio of 50:1. Consequently, the soaped dyed wool yarns were dried in an oven at 60 °C.

### Characterization

The chromatic values (L*, a*, b*, C*, h, and K/S) of dyed wool yarns were detected using a spectrophotometer (CHN-Spec CS-650A, Hangzhou Color Spectrum Technology Company, China) in the range of 350–760 nm of wavelength with 10 nm interval at 20 random positions. The mean of 20 detections was used to determine the chromatic values of each sample. The color difference (∆E) of dyed samples before and after treatment was calculated using Eq. (2), where subscripts a and b refer to those after and before treatment, respectively. The color uniformity of dyed wool yarn was represented by the standard deviation (σ) of the K/S values^40^. The wash colorfastness was evaluated using the ISO 105-C06:1997 (Test number: C2S). The light fastness of dyed wool yarn was tested using a Xenon Test Chamber (Q-SUN Xe-1, Q-LAB, USA) with irradiation for 24 h^41^. The color difference (∆E, Eq. 2) of the dyed sample before and after irradiation was used to assess the light fastness^42^.

2 $$\Delta E=\sqrt{{({L}_{b}^{*}-{L}_{a}^{*})}^{2}+{{(a}_{b}^{*}-{a}_{a}^{*})}^{2}+{{(b}_{b}^{*}-{b}_{a}^{*})}^{2}}$$

## Results and discussion

### Stability of natural dyes

**Figure 4:**
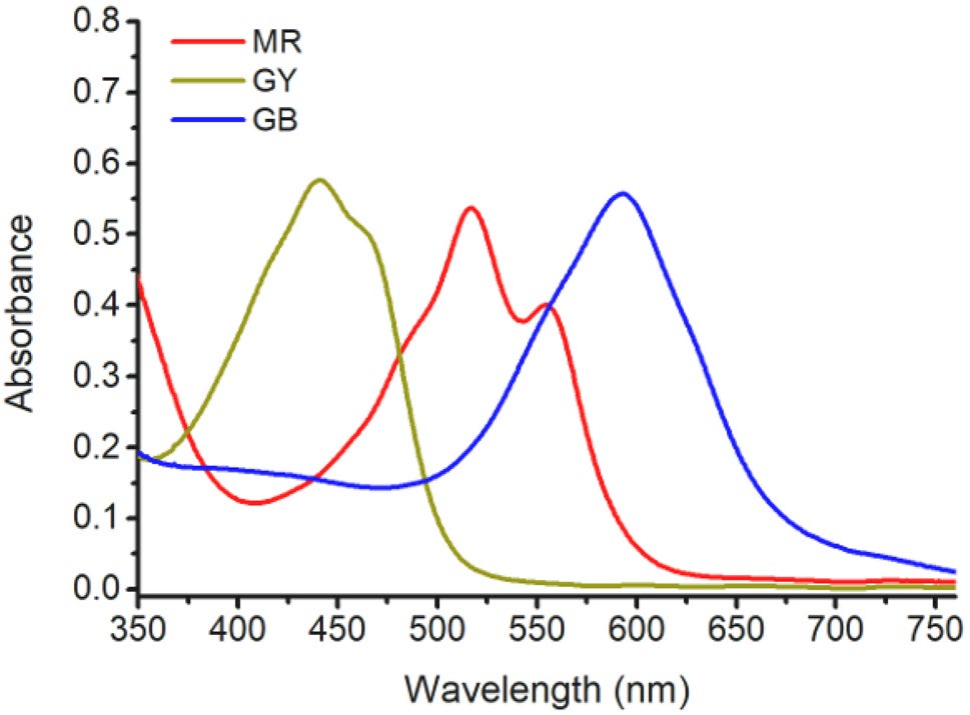
Light absorbance of MR, GB, and GY dye solutions.

The light absorbances of MR, GB, and GY dyes from 350 to 760 nm are shown in Fig. 4. The maximum absorbance wavelengths of MR dye (0.048 g L^−1^), GB dye (0.18 g L^−1^), and GY dye (0.1 g L^−1^) are 519 nm, 594 nm, and 441 nm, respectively. This indicates that at the same concentration of the used dyestuffs, the tinctorial strength of MR is the highest, followed by GY, and GB has the lowest tinctorial strength.

**Figure 5:**
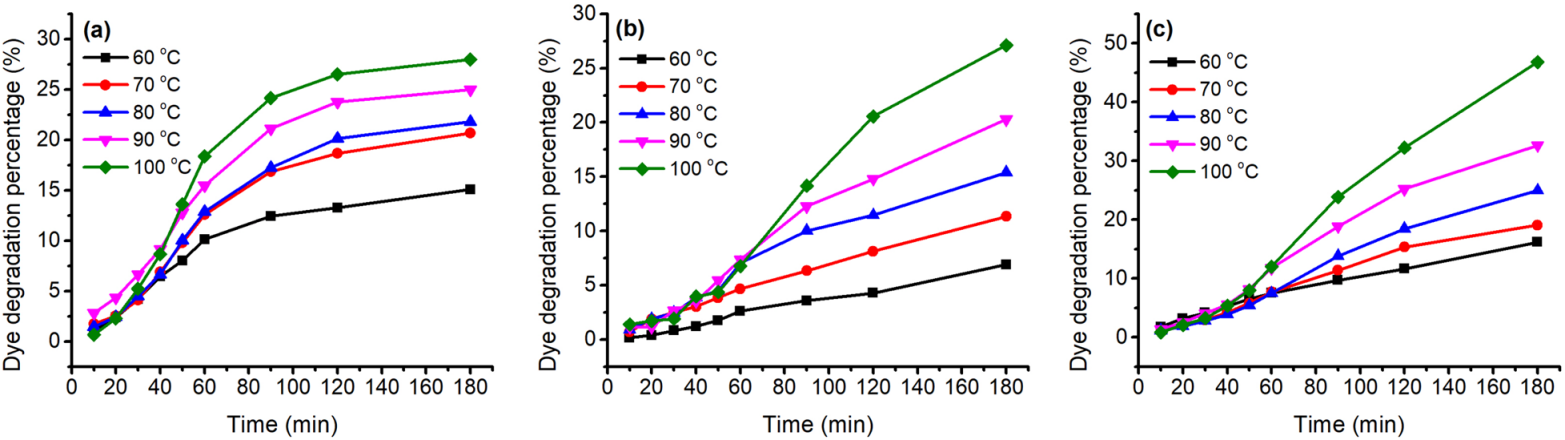
Thermostability of 80 mg L^−1^ of (**a**) MR, (**b**) GB, and (**c**) GY dyes in deionized water at pH 4 and 60–100 °C.

**Figure 6:**
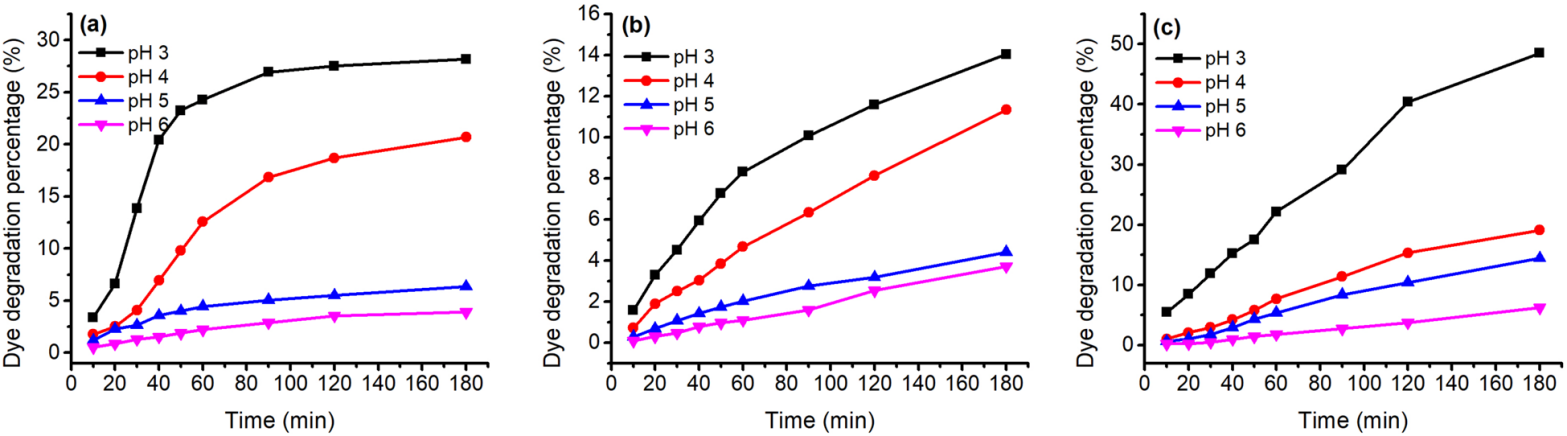
Thermostability of 80 mg L^−1^ of (**a**) MR, (**b**) GB, and (**c**) GY dyes in deionized water at pH 3–6 and 70 °C.

The stability of these three natural dyes was evaluated at various temperatures (60–100 °C), pH of dye solution (pH 3–6), and treatment time; their D% values are shown in Figs. 5 and 6. In the temperature variation study, the three natural dyes showed that the value of D% increased with an increase in temperature for these three dyes. This dye degradation tendency was also claimed in the case of cotton fabric dyeing with watermelon rind saps^43^. For 180 min treatment of GY dye, the D% values were 16.2, 19.1, 24.9, 32.6, and 46.8% for treating at 60, 70, 80, 90, and 100 °C, respectively. The other two dyes also exhibited a similar increasing tendency; the D% values of MR and GB increased from 15.1 to 28.0% and from 6.9 to 27.1% with increasing temperature from 60 to 100 °C for 180 min treatment, respectively. Besides, the D% values increased with an increase in treatment time for each treatment temperature^44^. These three natural dyes are sensitive to temperature, especially GY dye.

In the study of pH condition variation, the stability of these natural dyes decreased with a decrease in pH from pH 6 to pH 3, which is consistent with the previous study^45^. With the decrease in pH from pH 6 to pH 3, the D% values increased from 6.2 to 48.5% for GY dye (Fig. 6c), from 3.9 to 28.2% for MR dye (Fig. 6a), and from 3.7 to 14.0% for GB dye (Fig. 6b). Hence, the order of stability in pH sensitivity was GY > MR > GB. In addition, the D% almost linearly increased with the increase in treatment time for GB and GY dyes, while for MR dye, the increase in D% gradually reduced when the temperature reached the target temperature.

### Dyeing of wool yarn with a single natural dye

The results of the dye stability study indicate that during the dyeing, the natural dyes were partially degraded; hence, it is incorrect to use the light absorbance of the dye solution before and after dyeing to detect the dye amount absorbed in the wool yarn. Therefore, the description of the K/S values of dyed wool yarn is an alternative method to describe the real dye adsorption behavior in dyeing, and the higher K/S stands for, the higher dye adsorbed in wool yarn^46^.

**Figure 7:**
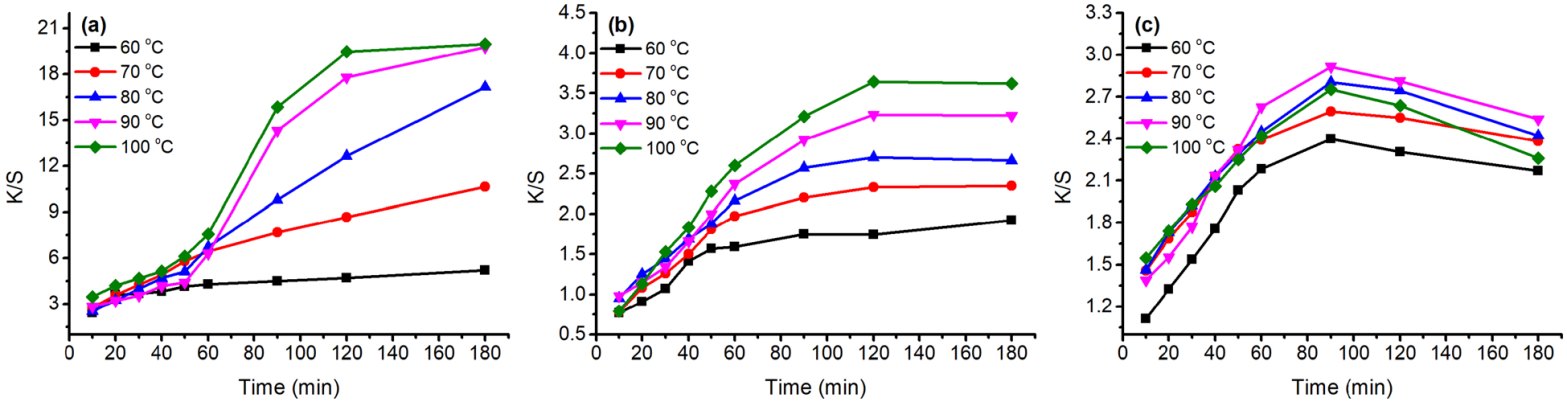
Color strength (K/S) of dyed wool fiber with 2% o.m.f of (**a**) MR, (**b**) GB, and (**c**) GY dyes in water at pH 4 and 60–100 °C.

The K/S values of dyed samples before soaping are shown in Figs. 7 and 9 with a variation in dyeing temperature (60–100 °C) and pH of the dye bath (pH 3–6), respectively. In the variation in dyeing temperature (Fig. 7), the K/S values increased with increasing dyeing temperature from 60 to 100 °C, except for the GY dyeing because its highest K/S was at 90 °C. The results can be attributed to the better swelling of fiber and dye migration in fiber at higher dyeing temperatures^47^. Thus, a higher dyeing temperature is beneficial for dye absorption in wool fiber.

Besides, after reaching the target temperatures, the dyes were still adsorbed in the wool yarns, except for the GY dye. In the dyeing with GY dye, after reaching the target temperatures, the dye was still adsorbed in the wool yarn first, but the GY dye mass decreased after 90 min of dyeing time in all dyeing temperatures, especially for the dyeing at 100 °C, which reduced to 2.3 at 180 min from 2.8 at 90 min dyeing time. The dramatic dye degradation can be attributed to the decrease in the K/S value. The dye degraded in the dye bath broke the previous dye adsorption balance, resulting in the dye adsorbed in wool fiber being desorbed and transferred to the dye bath. Therefore, the optimum dyeing temperature and dyeing time for GY were 90 °C and 90 min, respectively.

In the dyeing with MR dye, the dyeing equilibrium was 120 min after dyeing at 100 °C with a K/S of 19.5, and the K/S was 17.8 at 90 °C. In the dyeing with GB dye, the dye adsorptions were close to equilibrium for 120 min after dyeing. Hence, the optimum dyeing temperature and dyeing time for both MR and GB dyes were 100 °C and 120 min, respectively.

**Figure 8:**
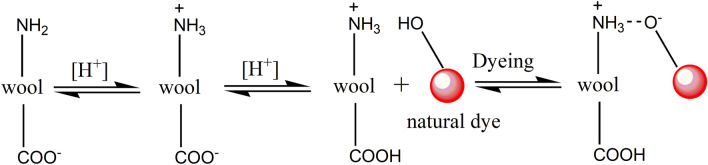
Mechanism of wool dyeing with natural dye in acidic conditions.

**Figure 9:**
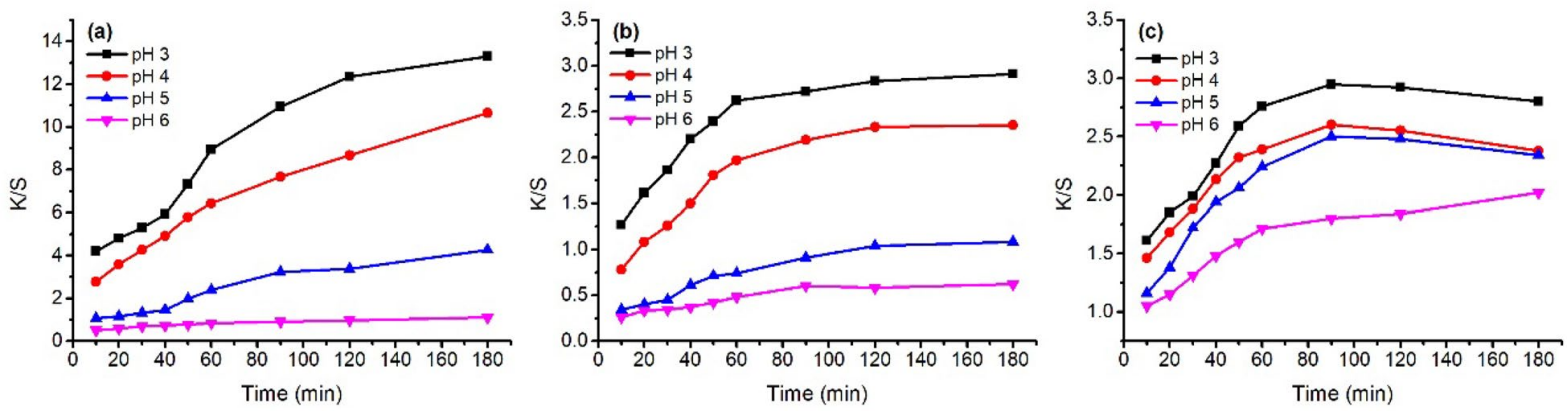
Color strength (K/S) of dyed wool fiber with 2% o.m.f of (**a**) MR, (**b**) GB, and (**c**) GY dyes in water at pH 3–6 at 70 °C.

In the variation of dye bath pH study, Fig. 9 shows that a decrease in dye bath pH was effective in promoting dye adsorption, i.e., K/S value for all these three dyes, which was proved by the previous study^48^. Wool fiber contained free carboxylic acid (–COOH) and amino (–NH_2_) groups; both groups exist in the zwitterion form when the wool is in water^49^. Thus, the lower the pH of the medium in which wool was present, the more the generated terminal amino groups^49^. Herein, the natural dyes were used as acidic dyes, forming ionic bonds with wool fiber; to be exact, the ionic bond between the anionic group of natural dye and the cationic terminal amino group of wool (Fig. 8). The cationic sites of terminal amino groups decreased with the increase in the pH of the dyeing medium^50^, and decreased the dye adsorption during dyeing, i.e., lowering the K/S value of dyed wool yarn.

In the dyeing with MR dye, the K/S values increased with increasing dyeing time under various pH conditions. Whereas, in the dyeing with GB dye, the K/S values increased for 60 min and then slightly increased in the last period of dyeing for pH 3 and 4 of the dye bath. In the dyeing with GY dye, the maximum K/S values for pH 3 to pH 5 were at 90 min after dyeing, with a slight decrease in the last period of dyeing, which can be ascribed to intensive degradation. Figure 9 shows that the highest dye adsorption present in the dyeing at pH 3 hinted that the partial dye degradation during dyeing might not influence the tendency of dyeing performance.

### Dyeing of wool yarn with natural dye mixtures

**Figure 10:**
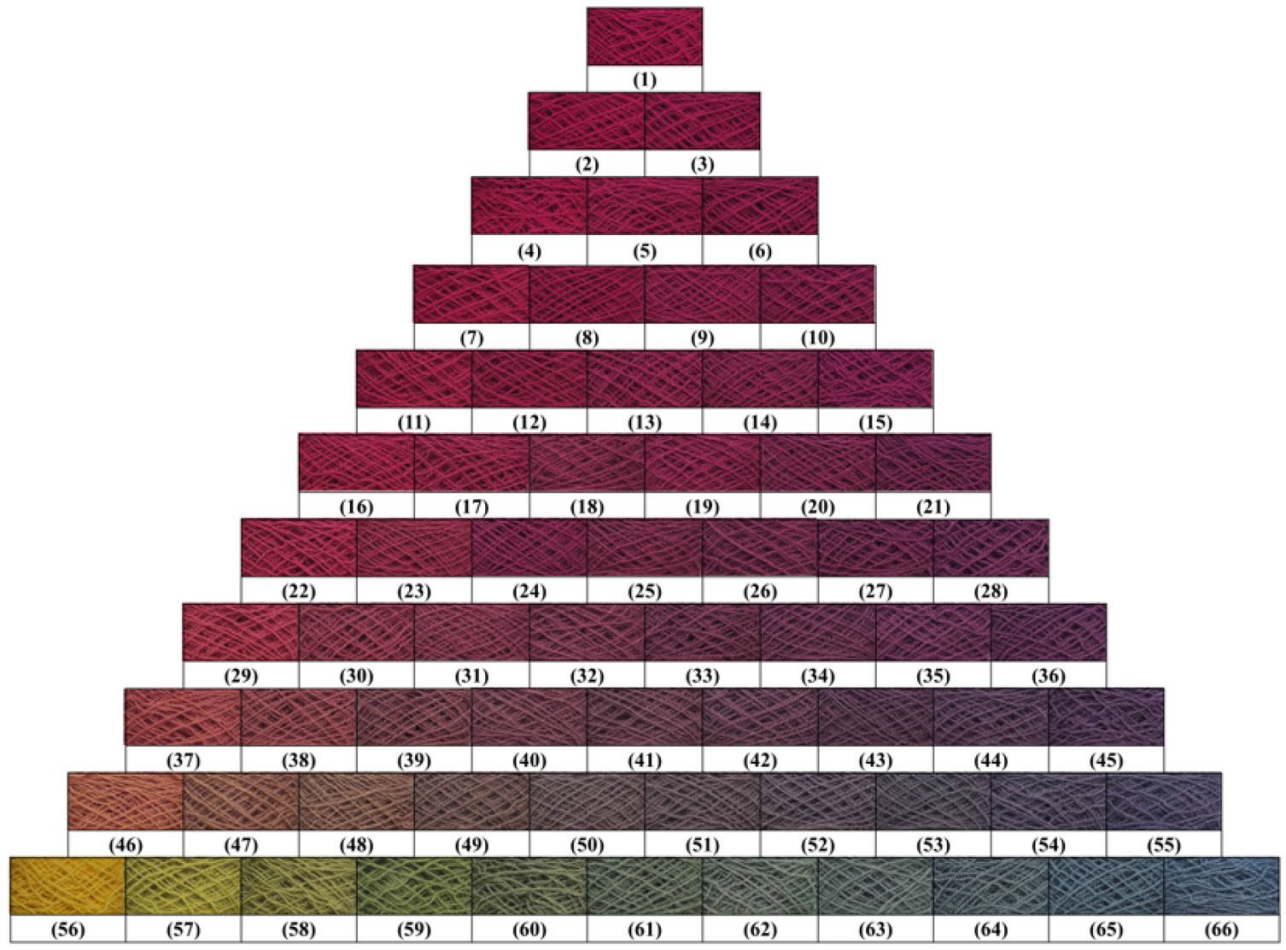
Dyed wool yarn with ternary mixtures of MR, GY, and GB before soaping.

**Figure 11:**
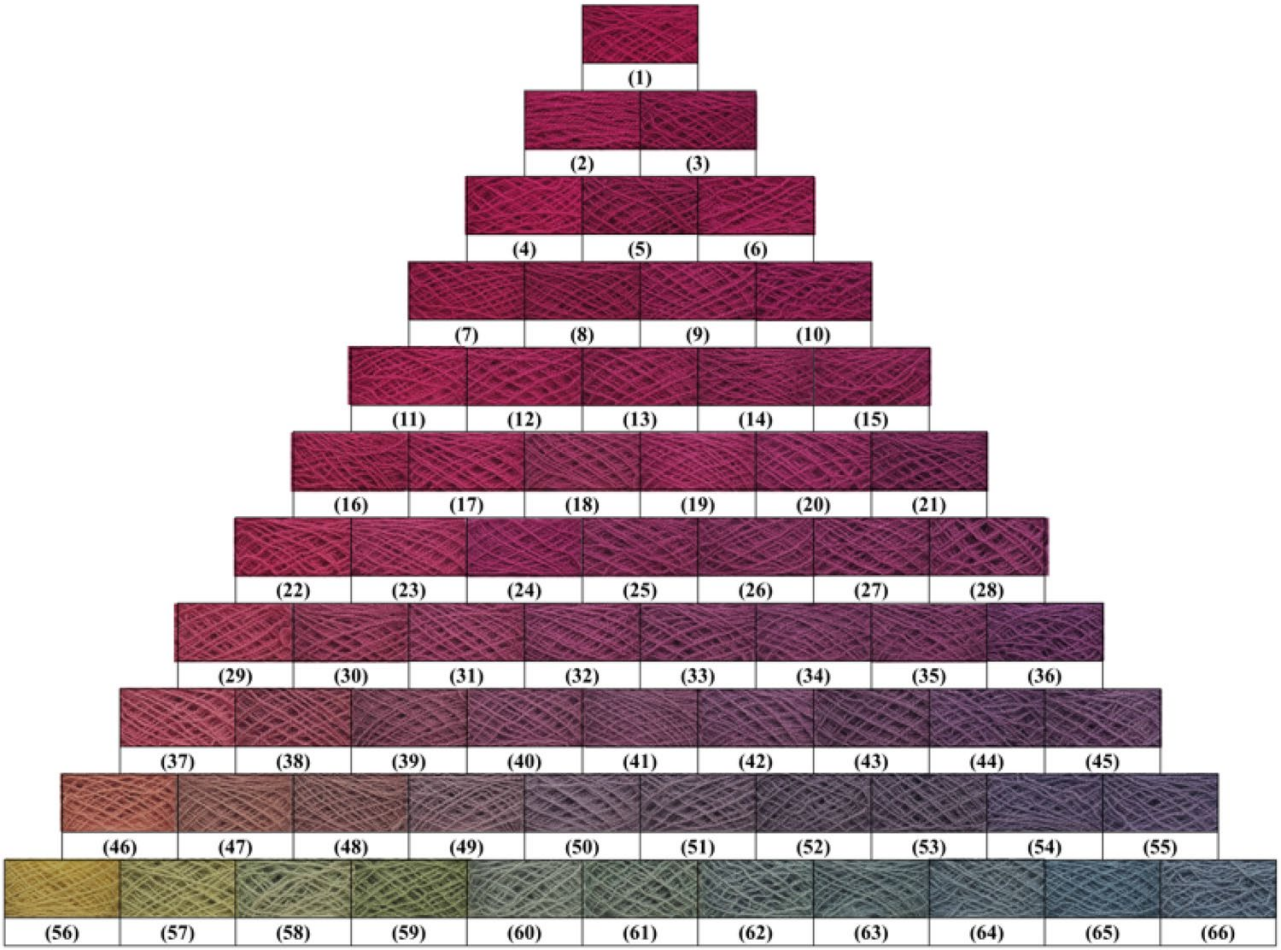
Dyed wool yarn with ternary mixtures of MR, GY, and GB after soaping.

The dyed wool yarns with binary and ternary natural dye mixtures before and after soaping are shown in Figs. 10 and 11, respectively. The chromatic values, K/S values, and dye fixation rates of wool yarns dyed with binary dye mixtures before and after soaping are shown in Tables 1, 2, and 3, and those with ternary dye mixtures are shown in Table 4.

**Table 1:** Chromatic values of wool yarns dyed with binary mixtures of MR and GY.

Sample number^※^	Dye mass ratio		Chromatic value						λ_max_	K/S
	MR	GY	L*	a*	b*	∆E	C*	h (^o^)		
1	1	0	30.3	40.3	2.3	4.2	40.3	3.2	530	21.8
1^#^			34.2	40.5	0.6		40.5	0.8	530	16.2
2	0.9	0.1	30.8	37.5	1.9	3.9	37.5	3.0	530	18.2
2^#^			33.7	38.0	− 0.6		38.0	359.0	530	15.2
4	0.8	0.2	31.7	38.2	3.4	4.5	38.3	5.1	530	17.9
4^#^			35.2	39.4	0.8		39.4	1.1	530	14.6
7	0.7	0.3	32.5	36.3	3.6	3.4	36.5	5.6	530	14.7
7^#^			33.9	35.4	0.6		35.4	1.0	530	13.0
11	0.6	0.4	32.5	35.5	5.2	5.8	35.9	8.4	530	14.9
11^#^			36.4	36.4	1.1		36.4	1.7	530	11.8
16	0.5	0.5	34.5	32.6	5.9	4.3	33.1	10.2	530	11.1
16^#^			36.4	32.5	2.0		32.5	3.6	530	9.8
22	0.4	0.6	37.7	30.7	7.2	5.9	31.6	13.2	530	8.3
22^#^			40.8	32.4	2.5		32.5	4.4	530	7.3
29	0.3	0.7	40.2	27.1	10.7	5.1	29.1	21.5	530	6.3
29^#^			41.9	27.2	5.8		27.8	12.0	530	5.7
37	0.2	0.8	44.0	21.5	14.2	8.4	25.8	33.5	530	4.3
37^#^			46.9	23.7	6.7		24.6	15.7	530	3.8
46	0.1	0.9	50.4	15.3	21.8	5.4	26.6	55.0	450	4.4
46^#^			49.8	15.4	16.4		22.5	46.9	450	3.7
56	0	1	71.9	5.3	52.2	18.5	52.5	84.3	450	3.9
56^#^			73.4	1.0	34.3		34.3	88.3	450	1.7

^※^Sample number without and with^#^ signal indicates the dyed sample before and after soaping, respectively.

**Table 2:** Chromatic values of wool yarns dyed with binary mixtures of MR and GB.

Sample number^※^	Dye mass ratio		Chromatic value						λ_max_	K/S
	MR	GB	L*	a*	b*	∆E	C*	h (^o^)		
1	1	0	30.3	40.3	2.3	4.2	40.3	3.2	530	21.8
1^#^			34.2	40.5	0.6		40.5	0.8	530	16.2
3	0.9	0.1	29.5	35.2	− 0.8	4.1	35.2	358.7	530	18.1
3^#^			30.2	31.6	− 2.7		31.7	355.1	530	15.5
6	0.8	0.2	29.4	33.1	− 2.2	5.8	33.1	356.2	530	17.2
6^#^			34.5	35.8	− 2.8		35.9	355.6	530	13.0
10	0.7	0.3	29.2	29.9	− 3.7	3.8	30.1	352.9	530	15.8
10^#^			32.4	31.3	− 5.2		31.8	350.6	530	12.7
15	0.6	0.4	30.3	28.5	− 6.0	5.5	29.1	348.2	530	13.4
15^#^			34.9	31.5	− 5.5		32.0	350.0	530	10.7
21	0.5	0.5	30.1	23.8	− 6.9	2.4	24.8	343.9	530	11.9
21^#^			31.8	25.1	− 8.1		26.4	342.2	530	11.2
28	0.4	0.6	30.9	20.6	− 8.6	6.3	22.3	337.4	530	10.1
28^#^			35.8	24.5	− 8.8		26.0	340.3	530	8.2
**36**	0.3	0.7	32.8	15.0	− 9.3	4.1	17.6	328.2	**570**	7.4
**36**^**#**^			35.8	17.1	− 11.1		20.3	327.0	**570**	6.4
45	0.2	0.8	34.3	10.0	− 11.2	5.4	15.0	311.7	570	6.5
45^#^			39.5	11.5	− 11.0		15.9	316.2	570	4.7
55	0.1	0.9	38.7	3.1	− 11.6	2.5	12.0	284.8	600	5.0
55^#^			40.8	4.4	− 11.5		12.3	290.8	580	4.2
66	0	1	42.3	− 7.4	− 11.5	5.8	13.7	237.1	610	5.4
66^#^			47.7	− 7.6	− 9.6		12.2	231.7	610	3.5

Significant values are in [bold].

^※^Sample number without and with^#^ signal mean the dyed sample before and after soaping, respectively.

**Table 3:** Chromatic values of wool yarns dyed with binary mixtures of GY and GB.

Sample number^※^	Dye mass ratio		Chromatic value						λ_max_	K/S
	GY	GB	L*	a*	b*	∆E	C*	h (^o^)		
56	1	0	71.9	5.3	52.2	18.5	52.5	84.3	450	3.9
56^#^			73.4	1.0	34.3		34.3	88.3	450	1.7
57	0.9	0.1	63.0	− 7.3	35.1	13.1	35.8	101.7	450	3.6
57^#^			64.7	− 6.3	22.2		23.0	105.9	450	1.9
58	0.8	0.2	56.2	− 8.5	24.9	11.5	26.3	108.8	450	3.7
58^#^			60.7	− 7.8	14.3		16.3	118.5	450	1.8
59	0.7	0.3	53.6	− 10.4	19.2	4.0	21.8	118.4	450	3.5
59^#^			55.6	− 10.3	15.7		18.8	123.1	450	2.7
60	0.6	0.4	51.2	− 10.8	13.4	9.2	17.2	128.8	450	3.2
60^#^			55.0	− 9.3	5.2		10.6	150.8	450	1.9
61	0.5	0.5	48.5	− 11.2	8.5	3.4	14.0	142.8	450	3.1
61^#^			50.6	− 10.4	6.0		12.0	150.0	610	2.6
62	0.4	0.6	48.7	− 11.1	5.4	5.6	12.3	154.0	610	3.1
62^#^			51.2	− 9.6	0.6		9.6	176.7	610	2.6
63	0.3	0.7	46.1	− 10.2	0.8	3.6	10.2	175.4	610	3.7
63^#^			48.5	− 10.2	− 1.9		10.4	190.5	610	3.3
64	0.2	0.8	43.9	− 9.5	− 3.7	4.0	10.2	201.5	610	4.5
64^#^			47.4	− 8.7	− 5.3		10.1	211.4	610	3.5
65	0.1	0.9	44.4	− 8.8	− 7.1	1.1	11.3	219.0	610	4.5
65^#^			45.4	− 8.7	− 7.6		11.5	221.0	610	4.2
66	0	1	42.3	− 7.4	− 11.5	5.8	13.7	237.1	610	5.4
66^#^			47.7	− 7.6	− 9.6		12.2	231.7	610	3.5

^※^Sample number without and with^#^ signal mean the dyed sample before and after soaping, respectively.

**Table 4:** Chromatic values of wool yarns dyed with ternary mixtures of MR, GY, and GB.

Sample number^※^	Dye mass ratio			Chromatic value						λ_max_	K/S
	MR	GY	GB	L*	a*	b*	∆E	C*	h (^o^)		
5	0.8	0.1	0.1	29.7	33.4	0.0	3.0	33.4	0.0	530	17.0
5^#^				31.8	33.4	− 2.2		33.4	356.2	530	14.3
8	0.7	0.2	0.1	30.3	33.2	0.6	3.3	33.2	1.0	530	16.1
8^#^				32.4	31.9	− 1.7		32.0	357.0	530	13.1
9	0.7	0.1	0.2	29.5	30.9	− 1.4	5.0	30.9	357.4	530	16.2
9^#^				33.5	33.5	− 3.0		33.6	354.9	530	12.9
12	0.6	0.3	0.1	31.7	34.2	1.8	6.0	34.3	3.1	530	16.4
12^#^				36.9	33.9	− 1.1		34.0	358.1	530	10.1
13	0.6	0.2	0.2	31.3	30.2	− 0.5	4.2	30.2	359.1	530	13.7
13^#^				34.6	31.1	− 2.7		31.2	355.0	530	11.0
14	0.6	0.1	0.3	31.4	28.9	− 2.9	3.8	29.0	354.3	530	12.7
14^#^				34.2	31.0	− 4.4		31.3	351.9	530	11.2
17	0.5	0.4	0.1	33.5	30.2	2.7	6.0	30.3	5.2	530	11.3
17^#^				37.8	32.8	− 0.5		32.8	359.2	530	9.1
18	0.5	0.3	0.2	34.5	25.6	1.2	5.1	25.6	2.6	530	9.0
18^#^				37.7	26.8	− 2.6		26.9	354.5	530	7.6
19	0.5	0.2	0.3	31.7	28.0	− 0.4	7.4	28.0	359.2	530	12.0
19^#^				37.8	31.0	− 3.2		31.2	354.1	530	8.4
20	0.5	0.1	0.4	31.6	25.3	− 4.5	5.4	25.6	350.0	530	11.3
20^#^				36.0	27.9	− 5.8		28.5	348.3	530	9.0
23	0.4	0.5	0.1	35.8	27.8	4.1	6.4	28.1	8.4	530	8.9
23^#^				40.1	30.2	0.0		30.2	359.9	530	7.2
24	0.4	0.4	0.2	31.6	26.8	− 3.0	3.4	26.9	353.5	530	11.5
24^#^				34.4	28.0	− 4.5		28.4	350.8	530	10.1
25	0.4	0.3	0.3	32.3	22.6	− 1.5	6.1	22.7	356.2	530	9.8
25^#^				36.9	24.8	− 4.8		25.3	349.1	530	7.5
26	0.4	0.2	0.4	32.7	22.2	− 4.4	4.3	22.6	348.8	530	9.2
26^#^				35.9	23.9	− 6.7		24.8	344.4	530	7.9
27	0.4	0.1	0.5	31.8	21.3	− 6.7	6.3	22.3	342.5	530	9.7
27^#^				37.1	24.6	− 7.5		25.7	343.2	530	7.5
30	0.3	0.6	0.1	37.8	22.1	5.3	5.2	22.7	13.5	530	6.5
30^#^				40.6	23.5	1.1		23.6	2.7	530	5.7
31	0.3	0.5	0.2	35.6	19.8	3.0	9.0	20.0	8.7	530	7.2
31^#^				41.9	23.3	− 2.4		23.4	354.2	530	5.1
32	0.3	0.4	0.3	34.9	17.9	0.2	7.5	17.9	0.5	530	7.1
32^#^				40.0	20.9	− 4.4		21.4	348.2	530	5.5
33	0.3	0.3	0.4	34.2	17.0	− 2.7	6.4	17.2	350.9	530	7.2
33^#^				38.8	19.6	− 6.3		20.6	342.0	530	5.7
34	0.3	0.2	0.5	33.8	16.5	− 4.9	6.3	17.2	343.5	530	7.3
34^#^				38.6	19.1	− 7.8		20.6	337.7	530	5.7
35	0.3	0.2	0.5	33.8	16.5	− 4.9	6.3	17.2	343.5	530	7.3
35^#^				38.6	19.1	− 7.8		20.6	337.7	530	5.7
38	0.2	0.7	0.1	41.6	17.4	10.5	6.9	20.3	31.0	460	4.9
38^*#*^				44.5	19.0	4.4		19.5	13.0	530	3.9
39	0.2	0.6	0.2	39.0	12.9	5.3	5.4	13.9	22.5	480	4.9
39^*#*^				41.3	14.1	0.6		14.1	2.4	530	4.3
40	0.2	0.5	0.3	38.4	12.3	2.4	7.6	12.5	11.1	530	4.8
40^#^				42.8	14.5	− 3.4		14.8	346.7	530	3.9
41	0.2	0.4	0.4	37.3	10.6	− 0.1	6.8	10.6	359.3	570	5.0
41^#^				41.2	13.0	− 5.2		14.0	338.3	570	4.2
42	0.2	0.3	0.5	36.7	10.3	− 2.1	6.1	10.5	348.5	570	5.3
42^#^				40.4	12.1	− 6.6		13.8	331.5	570	4.3
43	0.2	0.2	0.6	35.0	9.6	− 4.8	5.7	10.7	333.3	570	6.0
43^#^				39.3	12.0	− 7.7		14.3	327.5	570	4.7
44	0.2	0.1	0.7	34.9	9.1	− 7.6	4.6	11.8	319.9	570	6.1
44^#^				38.1	11.3	− 10.2		15.2	317.9	570	5.2
47	0.1	0.8	0.1	46.2	8.9	15.0	5.3	17.4	59.5	460	4.5
47^#^				48.1	9.6	10.2		14.0	46.6	450	3.2
48	0.1	0.7	0.2	45.6	5.5	11.8	5.5	13.0	65.3	460	4.1
48^#^				47.3	7.3	6.9		10.1	43.4	450	2.9
49	0.1	0.6	0.3	43.3	4.3	7.2	7.6	8.4	59.5	450	3.9
49^#^				47.5	6.4	1.3		6.6	11.2	570	2.5
50	0.1	0.5	0.4	41.4	3.1	3.4	8.2	4.6	48.1	450	3.8
50^#^				47.0	5.7	− 1.9		6.0	341.1	570	2.6
51	0.1	0.4	0.5	41.2	2.8	0.8	6.3	2.9	15.3	580	3.7
51^#^				45.1	5.0	− 3.7		6.2	-36.4	570	3.0
52	0.1	0.3	0.6	39.4	2.0	− 1.8	4.1	2.7	317.1	600	4.3
52^#^				40.7	3.3	− 5.5		6.4	300.8	580	4.1
53	0.1	0.2	0.7	39.6	2.0	− 5.1	3.5	5.5	291.2	600	4.4
53^#^				41.7	3.3	− 7.6		8.3	293.4	580	3.9
54	0.1	0.1	0.8	39.8	2.1	− 8.2	2.2	8.5	284.5	600	4.5
54^#^				40.6	3.1	− 10.0		10.4	287.2	580	4.3

^※^Sample number without and with^#^ signal mean the dyed sample before and after soaping, respectively.

In the dyeing with binary mixtures, the color shades of dyed samples before and after soaping regularly changed due to the inerratic change in dye mass ratio. As shown in Table 1, with a decrease in MR and an increase in GY mass ratios in mixtures among the dyed samples before soaping, a decreased tendency of a* values of CIE lab color space and increased tendencies of L* and b* values of CIE lab color space is presented, because MR (red color) mainly contributes to the positive a* value, while GY (yellow color) mainly affects the positive b* value, according to the color coordinate.

In Table 1, the dye mass ratio was 0.2:0.8 (MR:GY) for Sample 37. However, the λ_max_ of K/S was 530 nm, and h was 33.5°, indicating that the main color hue of the sample is reddish. The photograph of Sample 37 in Fig. 10 also identified the color hue. It was possibly caused by the dye mass adsorbed in the wool fiber and the tinctorial strength of dyes. The results of the thermostability study of these three natural dyes clearly show that these three dyes degraded at 90 °C (Fig. 5) and pH 3 (Fig. 6). Thus, the natural dyes were adsorbed in wool yarn during dyeing and suffered from degradation simultaneously. Notably, the dye concentration during the dyeing was reduced more dramatically than in the dye degradation treatment because of the dye adsorption in wool fiber, i.e., the dye degradation percentage in dyeing was less than in degradation.

**Figure 12:**
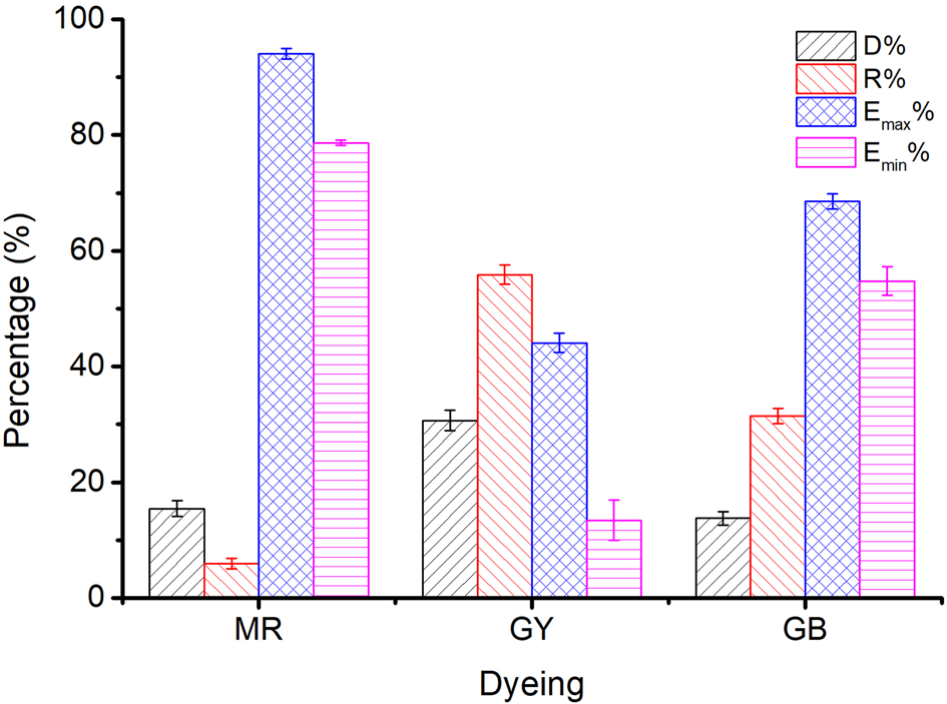
Dye residual percentage (R%), degraded percentage (D%), and the maximum and minimum dye exhaustion percentages (Emax% and Emin%) in dyeing with MR (Sample 1), GY (Sample 56), and GB (Sample 66).

Figure 12 shows the results of dyeing with MR (Sample 1), GY (Sample 56), and GB (Sample 66) and their respective control dyeing (without the addition of wool yarn). The dye degradation percentage (D%), residual percentage (R%), maximum dye exhaustion percentage (E_max_%), and minimum dye exhaustion percentage (E_min_%) were calculated using Eqs. (1), (3)–(5), respectively.

**Figure 13:**
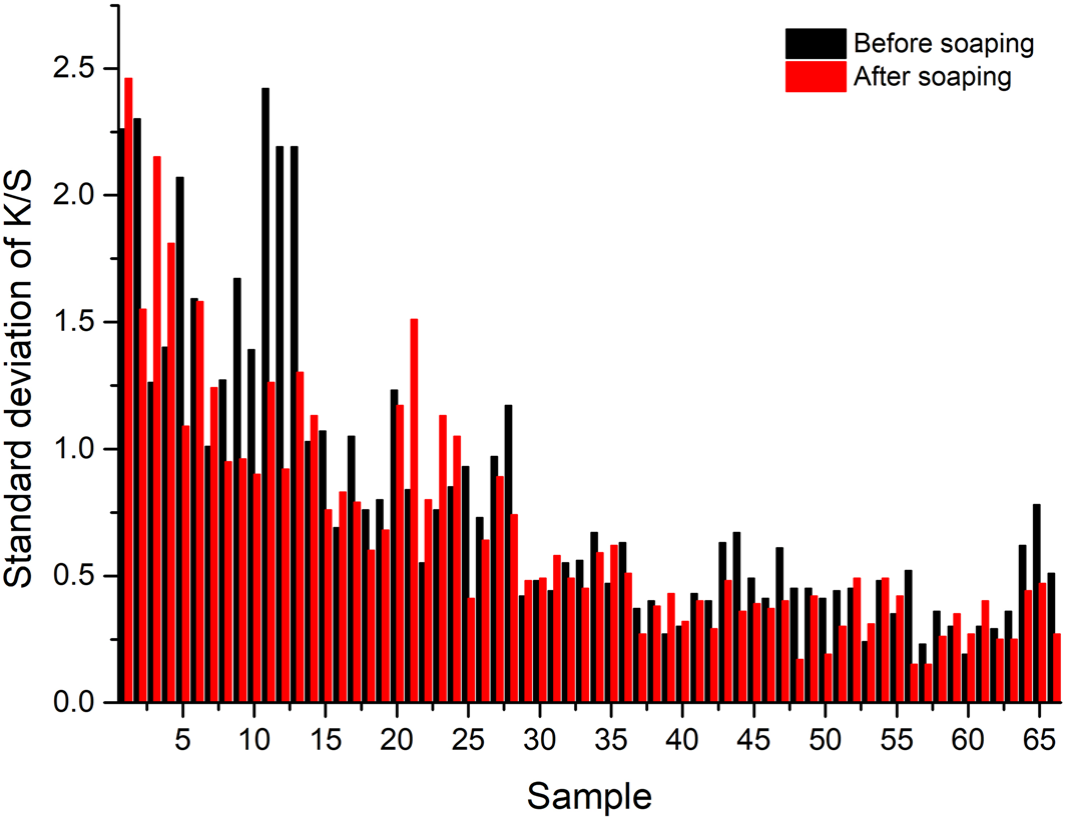
Standard deviation of K/S values of dyed wool yarns.

3 $$\mathrm{R\%}=\frac{{\mathrm{A}}_{2}}{{\mathrm{A}}_{0}}\times 100\mathrm{\%}$$

4 $${\mathrm{E}}_{\mathrm{max}}\mathrm{\%}=100\mathrm{\%}-\mathrm{R\%}$$

5 $${\mathrm{E}}_{\mathrm{min}}\mathrm{\%}=100\mathrm{\%}-\mathrm{R\%}-\mathrm{D\%}$$

where A_2_ is the light absorbance of the residual dye bath at λ_max_.

When the natural dyes did not degrade during dyeing, i.e., the D% was null, the E% was maximum (E_max_%); or during the dyeing of wool yarn, the tendency of dye degradation was similar to that in the control dyeing, i.e., the D% was maximum, so the E% was minimum (E_min_%). However, actually, partial natural dyes degraded during the wool fiber dyeing, and the dye adsorption in wool fiber reduced the amount of dye degradation in comparison to control dyeing. Thus, the E% should be within a range of E_min_% to E_max_%.

Figure 12 shows that the E% values of MR, GY, and GB were in the ranges of 78.7–94.1%, 13.4–44.1%, and 54.8–68.5%, respectively, and without overlapping. In other words, in the dyeing with a single dye, MR dyeing had the highest E%, GB dyeing was the next, and GY dyeing had the lowest E%, because of the highest R% and D% for GY. Thus the higher E% of MR than GY contributed to the reddish hue for Sample 37. Moreover, the GY has a lighter tinctorial strength than MR; hence, with the increased ratio of GY, the L* values increased. Besides, the maximum wavelength of the dyed samples’ K/S changed to 450 nm from 530 nm while the dye mass ratio was 0.1:0.9 (Sample 46), indicating that GY primarily contributed to the color shade.

In the description of color shade using the CIE Lch color space, the C* values (color saturation) generally decreased with the decrease in MR dye ratios for the dyed samples with or without soaping, indicating that MR primarily reduced the C* in the dyeing with binary mixtures of MR and GY dyes. C* value is dependent on the a* and b* values, and it is determined using Eq. (6). Because the higher E% and stronger tinctorial strength of MR than GY, the decrease of MR component in the binary mixture of MR and GY decreased the C* values. The h value was used to assess the hue of the sample. The maximum wavelength of K/S shifted to 450 nm from 530 nm, whereas the dye mass ratio was 0.1:09 (MR:GY), accompanied by an h value of 55.0° for the dyed sample before soaping, indicating that the dyed samples had more yellowness.

6 $$\text{C*} = \sqrt{{\text{a*}}^{2}+{\text{b*}}^{2}}$$

The color of the dyed sample changed by the soaping process, and the color difference (∆E) was within the range of 3.4–8.4 for the dyeing of wool yarns with binary mixtures of MR and GY dyes. The ∆E for the single MR dye was 4.2, while the ∆E for the single GY jumped to 18.5. Because the dyeing was executed at pH 3, an excessive GY was possibly adsorbed in wool yarn. Excessive adsorption usually occurs in the dyeing of wool with an acidic dye under strongly acidic conditions. Thus, the unfixed natural dyes in wool fiber were washed off after soaping, accompanied by color change. Owing to a temperature of 95 °C during soaping, it was impossible to detect the dye fixation rate by the light absorbance of the soaping solution because these natural dyes are easily degraded at high temperatures (Figs. 5 and 6). Besides, in the dyeing with binary mixtures of dyes, the dye amount removal ratio of each dye from the dyed wool yarn was occasionally different, for example, in Sample 55 in Table 2, shifting the λ_max_ of K/S after soaping. Furthermore, these natural plant dyes contain many compounds^51–54^, which are also adsorbed in the substance and contribute to the color shade of the dyed sample in its application. Hence, after soaping the wool yarn dyed with a single dye, the color shift was caused by the change in the amount of all the compounds in the wool yarn, although the λ_max_ of K/S was the same. Therefore, the dye fixation rate cannot be calculated from the ratio of the K/S value at the λ_max_ before soaping to that after soaping.

In the dyeing with binary MR and GB mixtures, the chromatic values of dyed wool yarns obtained before and after soaping are shown in Table 2. The chromatic values of L*a*b* show a similar change tendency in comparison with the dying using binary MR and GY mixtures. The blue color of GB contributed a negative b* value. Thus, with an increase in GB ratio in the mixture of MR and GB, the b* value of the dyed samples changed to a minus value, and its absolute value became larger, indicating that the GB dye was adsorbed more in the wool yarns. The λ_max_ of K/S was 530 nm for Sample 28 with a ratio of 0.4:0.6 (MR:GB). Then, the λ_max_ of K/S shifted to 570 nm at a ratio of 0.3:0.7 (Sample 36) and 600 nm at a ratio of 0.1:0.9 (Sample 55) due to the higher E% of GB. After soaping, the unfixed dye wash-off weakened the color strength and increased the lightness because the L* values of dyed samples were promoted.

The chromatic values of dyed wool yarns with binary mixtures of GY and GB are shown in Table 3. GY mainly contributed to positive b* value, while GB mainly contributed to negative b*. Hence, with the increase in GY ratio, the b* decreased and shifted to a minus value, and the L* values decreased. However, the a* values slightly fluctuated within the range of − 7.30 to − 11.17. Both of these decreases indicate that the color of the dye sample became darker with the increase in GB content. Notably, the a* value (5.25) of Sample 56 immediately changed to minus (− 7.30) at a ratio of 0.9:0.1, indicating that GB had a green tint and a vital effect on the a* value. Thus, Sample 56 showed slight greenish color (Fig. 10). The λ_max_ of K/S was 450 nm for Sample 61 with a ratio of 0.5:0.5 (MR:GB), and it changed to 610 nm for Sample 62 with a ratio of 0.4:0.6. It was caused by the higher amount of GB in the dyed wool yarn because of its high E% (Fig. 12) and high ratio in the binary mixtures, although the tinctorial strength of GY is higher than GB (Fig. 4). The main hue of the dyed sample did not shift by soaping, except for Sample 61 The λ_max_ of K/S changed to 610 nm from 450 nm because more amount of GY was washed off by the soaping, in contrast with GB. The ∆E values of Samples 56 and 66 were 18.5 and 5.8, respectively, indicating that the fixation property of GY was poorer than GB. This result supports the explanation of the λ_max_ of K/S shift in Sample 61 after soaping.

The chromatic values of dyed wool yarn with ternary mixtures of MR, GY, and GB are shown in Table 4. The different chromatic values of dyed samples after soaping indicate that wool yarns with many color shades were prepared with different dye mass ratios. The color shades are shown in Fig. 11. Besides, the ∆E values had a small change, indicating that the dye fixation properties of these dyes were acceptable.

### Color uniformity

The color uniformity of dyed wool yarns expressed by the standard deviation of K/S values is shown in Fig. 13. Generally, most of the standard deviation values are lower than 1.0 for the dyed samples (Figs. 10 and 11) before and after soaping, indicating that the color on the surface of dyed samples was even. Meanwhile, the soaping process was beneficial for the color uniformity of dyed samples, because the standard deviation of dyed samples was reduced after soaping. Therefore, it can be concluded that in the dyeing with binary and ternary dyes mixtures, the natural dyes were equally distributed in the wool yarns and exhibited uniform color.

### Colorfastness to washing and lighting

**Figure 14:**
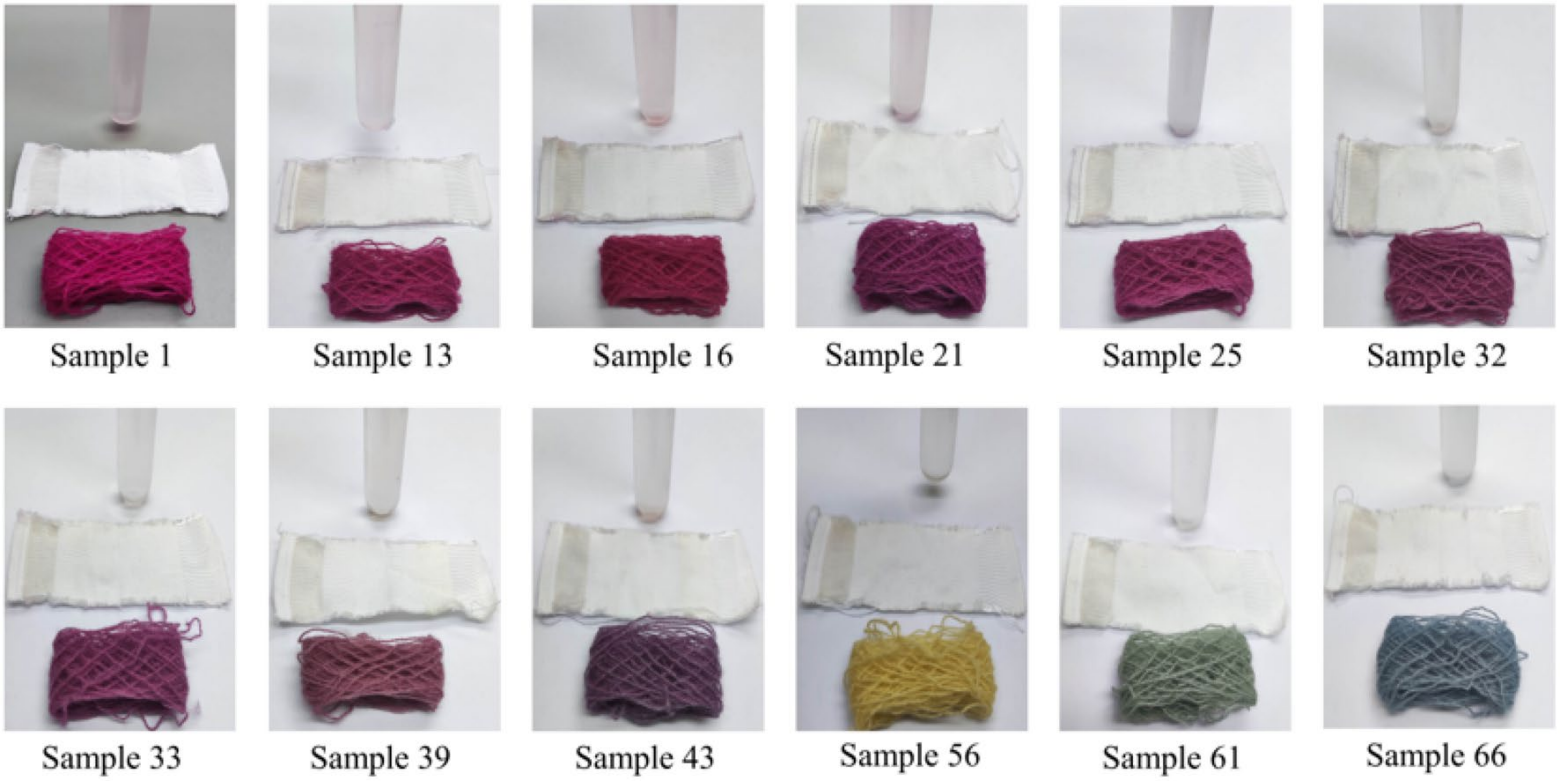
Wash colorfastness assessment of dyed wool yarns using singe dye, binary dye mixtures, and ternary dye mixtures.

The samples dyed with a single dye, binary dye mixture, and ternary dye mixture were selected to assess their wash colorfastness, as shown in Fig. 14. The wash colorfastness of these samples in staining the multifiber fabric was Grade 5 for all six fibers. Although the light color was present in the solution during testing, the multifiber fabric was clean without any staining, possibly because the testing conditions (pH 10.5) prevented the removal of dye from the multifiber. Furthermore, after testing, the fade color grade of wash colorfastness was found to be Grade 5 for all the tested samples. Therefore, the results of these selected samples indicate that the wash colorfastness of the dyed samples with a single dye and mixtures was Grade 5 for both the staining and fade colorfastness. Overall it shows that less amount of color is released during washing, which is very crucial for maintaining environmental sustainability.

**Figure 15:**
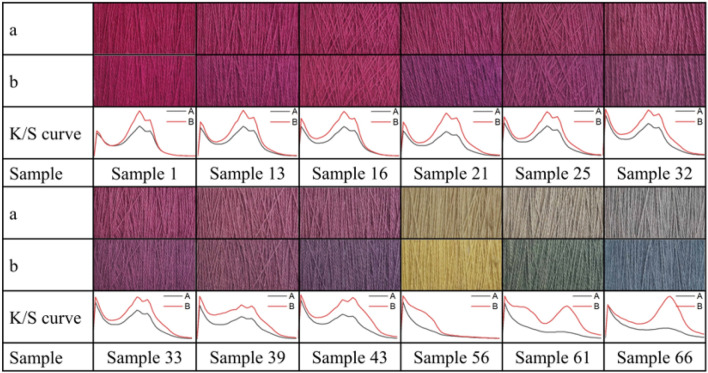
Photographs of dyed wool yarns (**a**) after and (**b**) before light colorfastness testing and their K/S curves.

The photographs of selected dyed wool yarns before and after light colorfastness testing are shown in Fig. 15, along with their K/S curves, and the color difference of dyed wool yarns before and after the light colorfastness testing is shown in Table 5. Undoubtedly, the colors of dyed wool yarns faded apparently due to the natural dye's inherent defect of unstable color by light irradiation^55^. After light irradiation, the K/S of dyed samples decreased, especially for GY and GB dyes. The color of the dyed sample with single GY (Sample 56) or GB (Sample 66) almost tended to be a grey color. In contrast, MR exhibited a relatively better light resistance^56^, although it also slightly faded after light irradiation. In the dyed wool yarn with binary and ternary mixtures, the MR component showed its vital contribution to light resistance. The color difference of dyed wool samples in Table 5 shows a significant trend of MR for light resistance because the ∆E of the dyed sample with MR was lower than that without the MR component (Samples 56, 61, and 66). Meanwhile, because the color faded, the L* values of all the samples increased.

**Table 5:** Color difference of dyed wool yarns before and after light colorfastness testing.

Sample^※^	L*	a*	b*	∆E	Sample	L*	a*	b*	∆E
1	34.2	40.5	0.6	5.4	33	38.8	19.6	− 6.3	8.0
1^#^	36.3	35.5	0.6		33^#^	45.4	22.2	− 2.5	
13	34.6	31.1	− 2.7	5.2	39	41.3	14.1	0.6	8.2
13^#^	39.5	30.2	− 1.3		39^#^	48.6	17.7	−** 0.2**	
16	36.4	32.5	2.0	5.6	43	39.3	12.0	− 7.7	11.2
16^#^	40.5	29.5	− 0.3		43^#^	48.0	16.3	− 2.2	
21	31.8	25.1	− 8.1	9.8	56	73.4	1.0	34.3	15.1
21^#^	39.8	26.9	− 2.7		56^#^	76.2	1.2	19.5	
25	36.9	24.8	− 4.8	6.7	61	50.6	− 10.4	6.0	18.5
25^#^	42.9	26.1	− 2.0		61^#^	65.8	− 0.9	10.4	
32	40.0	20.9	− 4.4	6.8	66	47.7	− 7.6	− 9.6	19.5
32^#^	45.9	23.1	− 1.9		66^#^	60.8	− 1.0	3.2	

^※^Sample number without and with^#^ signal mean the dyed sample before and after soaping, respectively.

## Conclusions

The sustainable dyeing of wool yarn in the presence of three representative natural dyes, MR, GB, and GY, was carried out without the addition of conventional mordants. Initially, the light absorbance data show that the tinctorial strength of MR is the highest, followed by GY and GB, which is also supported by their thermostability. In a single bath of natural dyeing, K/S values were measured to determine the real dye adsorption behavior, and it found the K/S values increased with increasing dyeing temperature from 60 °C to 100 °C, except for the GY dyeing because its highest K/S was at 90 °C. Then, a color triangle dyeing recipe (binary and ternary dyeing scheme) was formulated using around 66 samples; no overlapping tendency was obtained with the E% values of MR (78.7–94.1%), GY (13.4–44.1%), and GB (54.8–68.5%). Additionally, a color uniformity test confirmed that in the dyeing with binary and ternary dyes mixtures, the natural dyes were equally distributed in the wool yarns (uniform color), and the colorfastness properties were satisfactory in terms of less color reduction during the washing process especially. As a result, natural dye mixtures may be deemed as a suitable approach for coloring textile materials, with no problematic processes or negative environmental repercussions. Hence, in future research, attention will be paid to mixing natural colors to find natural dyes with green color and investigate their dyeing performance on wool fiber in mixtures, thereby expanding the range of colors for dyed wool yarn with a mixture of natural dyes.

## Data Availability

The datasets generated during the current study are available from the corresponding author on reasonable request (Prof. Yingjie Cai, Y. Cai).

## References

[1] Pervez MN, Balakrishnan M, Hasan SW, Choo K-H, Zhao Y, Cai Y, Zarra T, Belgiorno V, Naddeo V (2020). A critical review on nanomaterials membrane bioreactor (NMs-MBR) for wastewater treatment. NPJ Clean. Water.

[2] Pervez MN, Stylios GK, Liang Y, Ouyang F, Cai Y (2020). Low-temperature synthesis of novel polyvinylalcohol (PVA) nanofibrous membranes for catalytic dye degradation. J. Clean. Prod..

[3] Pervez MN, Telegin FY, Cai Y, Xia D, Zarra T, Naddeo V (2019). Efficient degradation of Mordant Blue 9 using the Fenton-activated persulfate system. Water.

[4] Hasan KMF, Pervez MN, Talukder ME, Sultana MZ, Mahmud S, Meraz MM, Bansal V, Genyang C (2019). A novel coloration of polyester fabric through green silver nanoparticles (G-AgNPs@PET). Nanomaterials.

[5] Morshed MN, Pervez MN, Behary N, Bouazizi N, Guan J, Nierstrasz VA (2020). Statistical modeling and optimization of heterogeneous Fenton-like removal of organic pollutant using fibrous catalysts: A full factorial design. Sci. Rep.-UK.

[6] Pervez MN, He W, Zarra T, Naddeo V, Zhao Y (2020). New sustainable approach for the production of Fe_3_O_4_/Graphene Oxide-activated persulfate system for dye removal in real wastewater. Water.

[7] Su S, Liang Y, Yin G, Wang Q, Cai Y, Peng X, Pervez MN, Lin L (2019). Anhydrous dyeing processes of ramie fiber in liquid ammonia. Cellulose.

[8] Hossain MY, Liang Y, Pervez MN, Ye X, Dong X, Hassan MM, Cai Y (2021). Effluent-free deep dyeing of cotton fabric with cacao husk extracts using the Taguchi optimization method. Cellulose.

[9] Habib MA, Pervez MN, Mahmud S, Khan MRM, Heng Q (2017). Macadamia integrifolia: A new source of natural dyes for textile colouration. Asian J. Chem..

[10] Hossain MY, Zhu W, Pervez MN, Yang X, Sarker S, Hassan MM, Hoque MIU, Naddeo V, Cai Y (2021). Adsorption, kinetics, and thermodynamic studies of cacao husk extracts in waterless sustainable dyeing of cotton fabric. Cellulose.

[11] Shabbir M, Rather LJ, Mohammad F (2018). Economically viable UV-protective and antioxidant finishing of wool fabric dyed with Tagetes erecta flower extract: Valorization of marigold. Ind. Crops Prod..

[12] Shafiq F, Siddique A, Pervez MN, Hassan MM, Naddeo V, Cai Y, Hou A, Xie K, Khan MQ, Kim I-S (2021). Extraction of natural dye from aerial parts of argy wormwood based on optimized taguchi approach and functional finishing of cotton fabric. Materials..

[13] Xu X, Gong J, Li Z, Li Q, Zhang J, Wang L, Huang J (2020). Mordant free dyeing and functionalization of wool fabrics with biocolorants derived from *Apocynum venetum* L. Bast. ACS. Sustain. Chem. Eng..

[14] Shahid M, Shahidul I, Mohammad F (2013). Recent advancements in natural dye applications: A review. J. Clean. Prod..

[15] Hossain MY, Jiang T, Zhu W, Sarker S, Pervez MN, Hoque MIU, Cai Y, Naddeo V (2022). Green and sustainable method to improve fixation of a natural functional dye onto cotton fabric using cationic dye-fixing agent/D5 microemulsion. J. Nat. Fibers.

[16] Shukla D, Vankar PS (2013). Natural dyeing with black carrot: New source for newer shades on silk. J. Nat. Fibers.

[17] Yi E, Cho JY (2008). Color analysis of natural colorant-dyed fabrics. Color. Res. Appl..

[18] Feiz M, Norouzi H (2014). Dyeing studies of wool fibers with madder (*Rubia tinctorum*) and effect of different mordants and mordanting procedures on color characteristics of dyed samples. Fiber. Polym..

[19] Telegin FY, Ran JH, Morshed M, Pervez MN, Sun L, Zhang C, Priazhinikova VG (2016). Structure and properties of dyes in coloration of textiles: Application of fragment approach. Key. Eng. Mat..

[20] Jahangiri A, Ghoreishian SM, Akbari A, Norouzi M, Ghasemi M, Ghoreishian M, Shafiabadi E (2018). Natural dyeing of wool by Madder (*Rubia tinctorum* L.) root extract using tannin-based biomordants: Colorimetric, fastness and tensile assay. Fiber. Polym..

[21] Burkinshaw SM, Kumar N (2009). The mordant dyeing of wool using tannic acid and FeSO_4_, Part 1: Initial findings. Dyes Pigments.

[22] Zheng GH, Fu HB, Liu GP (2011). Application of rare earth as mordant for the dyeing of ramie fabrics with natural dyes. Korean J. Chem. Eng..

[23] Cai Y, Liang Y, Navik R, Zhu W, Zhang C, Pervez MN, Wang Q (2020). Improved reactive dye fixation on ramie fiber in liquid ammonia and optimization of fixation parameters using the Taguchi approach. Dyes Pigments.

[24] Bechtold T, Turcanu A, Ganglberger E, Geissler S (2003). Natural dyes in modern textile dyehouses—how to combine experiences of two centuries to meet the demands of the future?. J. Clean. Prod..

[25] Glover B, Pierce JH (1993). Are natural colorants good for your health?. J. Soc. Dyers. Colour..

[26] Amutha K, Sudhapriya N (2020). Dyeing of textiles with natural dyes extracted from Terminalia arjuna and Thespesia populnea fruits. Ind. Crops. Prod..

[27] İşmal, Ö. E. & Yıldırım, L. In *The Impact and Prospects of Green Chemistry for Textile Technology* (eds Shahid ul, I. & Butola, B. S.) 57–82 (Woodhead Publishing, 2019).

[28] Samanta, A. K. & Konar, A. In *Natural Dyes* vol. 3 (ed Emriye, A. K.) 134 (IntechOpen, 2011).

[29] Mussak, R. A. & Bechtold, T. In *Handbook of Natural Colorants.* (eds Bechtold, T. & Mussak, R.A.M. ) 315–338 (Wiley, 2009).

[30] Mongkholrattanasit R, Kryštůfek J, Wiener J, Viková M (2011). UV protection properties of silk fabric dyed with eucalyptus leaf extract. J. Text..

[31] Shahidul I, Sun G (2017). Thermodynamics, kinetics, and multifunctional finishing of textile materials with colorants extracted from natural renewable sources. ACS. Sustain. Chem. Eng..

[32] Zhang Y, Shahidul I, Rather LJ, Li Q (2022). Recent advances in the surface modification strategies to improve functional finishing of cotton with natural colourants—a review. J. Clean. Prod..

[33] Samanta P, Singhee D, Samanta AK (2018). Fundamentals of natural dyeing of textiles: Pros and cons. Curr. Tren. Fash. Tech. Text. Eng..

[34] Derksen GCH, van Holthoon FL, Willemen HM, Krul CAM, Franssen MCR, van Beek TA (2021). Development of a process for obtaining non-mutagenic madder root (*Rubia tinctorum*) extract for textile dyeing. Ind. Crops Prod..

[35] Li W, Li J, Xu Y, Huang Y, Xu S, Ou Z, Long X, Li X, Liu X, Xiao Z, Huang J, Chen W (2021). Expression of heat-resistant β-glucosidase in *Escherichia coli* and its application in the production of gardenia blue. Synth. Syst. Biotechnol..

[36] Tsutsumiuchi K, Toyoshima T, Hasegawa F, Terasawa R, Honda W, Sakakibara M, Ishida Y, Ikai Y, Ishibashi R, Furuya K, Morimoto T, Ishizuki K, Nishizaki Y, Masumoto N, Sugimoto N, Sato K, Oka H (2021). Molecular structure of gardenia blue pigments by reaction of genipin with benzylamine and amino acids. J. Agric. Food Chem..

[37] Ozaki A, Kitano M, Furusawa N, Yamaguchi H, Kuroda K, Endo G (2002). Genotoxicity of gardenia yellow and its components. Food Chem. Toxicol..

[38] Cai Y, Su S, Navik R, Wen S, Peng X, Pervez MN, Lin L (2018). Cationic modification of ramie fibers in liquid ammonia. Cellulose.

[39] Zhang P, Zhang C, Jiang T, Hossain MY, Zhu W, Pervez MN, Hoque MIU, Khan I, Long X, Cai Y (2022). Dyeing of raw ramie yarn with Reactive Orange 5 dye. Ind. Crops. Prod..

[40] Lin L, Zhu W, Zhang C, Hossain MY, Oli ZBS, Pervez MN, Sarker S, Hoque MIU, Cai Y, Naddeo V (2021). Combination of wet fixation and drying treatments to improve dye fixation onto spray-dyed cotton fabric. Sci. Rep.-UK.

[41] Inamdar UY, Pervez MN, Navik RG, Peng X, Cai Y (2017). Low-temperature bleaching of cotton fabric by activated peroxide system. Emerg. Mater. Res..

[42] Liang Y, Zhu W, Zhang C, Navik R, Ding X, Mia MS, Pervez MN, Mondal MIH, Lin L, Cai Y (2021). Post-treatment of reactive dyed cotton fabrics by caustic mercerization and liquid ammonia treatment. Cellulose.

[43] Liman MLR, Islam MT, Hossain MM, Sarker P, Dabnath S (2021). Coloration of cotton fabric using watermelon extract: Mechanism of dye-fiber bonding and chromophore absorption. J. Text..

[44] Steet JA, Tong CH (1996). Degradation kinetics of green color and chlorophylls in peas by colorimetry and HPLC. J. Food. Sci..

[45] Ryan-Stoneham T, Tong CH (2000). Degradation kinetics of chlorophyll in peas as a function of pH. J. Food. Sci..

[46] Guesmi A, Hamadi NB, Ladhari N, Sakli F (2012). Dyeing properties and colour fastness of wool dyed with indicaxanthin natural dye. Ind. Crops. Prod..

[47] Zhang P, Zhu W, Hossain MY, Sarker S, Pervez MN, Mondal MIH, Yan C, Cai Y, Naddeo V (2022). Toward improved performance of reactive dyeing on cotton fabric using process sensitivity analysis. Int. J. Cloth. Sci. Tech..

[48] Kamel MM, Abdelghaffar F, El-Zawahry MM (2011). Eco-friendly dyeing of wool with a mixture of natural dyes. J. Nat. Fibers..

[49] Lewis DM, Rippon JA (2013). The Coloration of Wool and Other Keratin Fibres.

[50] Mehrparvar L, Safapour S, Sadeghi-Kiakhani M, Gharanjig K (2016). A cleaner and eco-benign process for wool dyeing with madder, *Rubia tinctorum* L., root natural dye. Int. J. Environ. Sci. Tech..

[51] Derksen GCH, van Holthoon FL, Willemen HM, Krul CAM, Franssen MCR, van Beek TA (2021). Development of a process for obtaining non-mutagenic madder root (*Rubia tinctorum*) extract for textile dyeing. Ind. Crops. Prod..

[52] Hobbs CA, Koyanagi M, Swartz C, Davis J, Maronpot R, Recio L, Hayashi S-M (2018). Genotoxicity evaluation of the naturally-derived food colorant, gardenia blue, and its precursor, genipin. Food. Chem. Toxicol..

[53] Kwon OO, Kim EJ, Lee JH, Kim TY, Park KH, Kim SY, Suh HJ, Lee HJ, Lee JW (2015). Photovoltaic performance of TiO_2_ electrode adsorbed with gardenia yellow purified by nonionic polymeric sorbent in dye-sensitized solar cells. Spectrochim. Acta. A..

[54] Yusuf M, Shahid M, Khan SA, Khan MI, Islam S-U, Mohammad F, Khan MA (2013). Eco-dyeing of wool using aqueous extract of the roots of indian Madder (*Rubia cordifolia*) as natural dye. J. Nat. Fibers..

[55] Yatagai, M., Magoshi, Y., Becker, M. A., Sano, C., Ikuno, H., Kohara, N. & Saito, M. In *Historic Textiles, Papers, and Polymers in Museums* vol. 779 *ACS Symposium Series* (eds Jeanette M. C. & Mary, T. B.) Ch. 7, 86–97 (American Chemical Society, 2000).

[56] Manhita A, Ferreira V, Vargas H, Ribeiro I, Candeias A, Teixeira D, Ferreira T, Dias CB (2011). Enlightening the influence of mordant, dyeing technique and photodegradation on the colour hue of textiles dyed with madder—a chromatographic and spectrometric approach. Microchem. J..

